# Clinical Trial Evaluating Quality of Life in Patients with Intra-Oral Halitosis

**DOI:** 10.3390/jcm11020326

**Published:** 2022-01-10

**Authors:** Iwona Olszewska-Czyz, Sarkis Sozkes, Agata Dudzik

**Affiliations:** 1 Periodontology, Prophylaxis and Oral Pathology Department, Medical Faculty, Jagiellonian University, 31155 Krakow, Poland; agata.skrzypek@uj.edu.pl; 2 Biomaterials Department, Biomedical Engineering Corlu Faculty, Tekirdag Namik Kemal University, 59860 Tekirdag, Turkey; ssozkes@nku.edu.tr

**Keywords:** halitosis, quality of life, malodor

## Abstract

Halitosis is considered to be extremely unattractive in the context of social interactions. The main research objective of this study was to evaluate whether intra-oral halitosis may impact patients’ quality of life (QOL). One hundred generally healthy adult participants complaining about oral malodor and diagnosed with intra-oral halitosis were enrolled in this study. For halitosis diagnosis, a gas chromatography (GC) analysis by the Oral Chroma portable device was used. QOL assessment was based on the Short Form 36-item Health Survey (SF-36). The respondents had the highest scores in the physical functioning (PF), activity limitations caused by emotional problems (RE) and activity limitations caused by physical problems (RP) domains, and the weakest in the general health perception (GH), vitality (VT) and emotional wellbeing (MH) ones. The total volatile sulfur compounds (VSCs) level was negatively correlated with SF-36 domains. The SF-36 domains’ scores decreased the higher the level of VSC was. The respondents assessed their QOL to be at its best in physical functioning and activity limitations caused by emotional and physical problems and the worst in general health perception, vitality and emotional wellbeing. The strongest correlation between halitosis and decreased QOL was found in the social functioning (SF), vitality, emotional wellbeing and general health perception domains.

## 1. Introduction

### 1.1. Background

Halitosis is a common complaint and a large concern of up to one-third of the general population [1]. The term halitosis describes a condition that refers to an unpleasant oral odor, which can be also called malodor, fetor ex ore or “bad breath” which can be defined as a socially unacceptable level of breath odor [2]. It is considered to be extremely unattractive in the context of social interactions; thus, individuals with halitosis often experience psychological consequences and poor quality of life (QOL) [3].

The nature of halitosis is multifactorial as the potential emanation of odor via the mouth and nose can come from the respiratory and gastroesophageal tracts, can be the result of the transfer of volatiles from blood to breath during alveolar gas exchange, and it may be also a result of the patient’s self-perception (bad breath paradox). The lack of clear boundary between physiologic breath odor and pathologic halitosis makes the medical approaches even more confusing, thus the diagnostic process should include not only anamnesis, oral examination and breath odor measurements but also the patient’s perception and their psychological investigation [4]. 

The most common classification of halitosis was introduced by Miyazaki et al. [5]. It divides the condition into: genuine halitosis (physiologic or pathologic: intra-oral and extra-oral), pseudohalitosis and halitophobia; however, according to some authors, pseudohalitosis and halitophobia classifications are inadequate [6]. When diagnosing and treating patients with halitosis, it is important to distinguish between different types and potential etiological factors; Aydin and Harvey-Woodworth have thus suggested a new approach and classified the condition into types: 0—physiologic halitosis; 1—oral halitosis; 2—airway halitosis; 3—gastroesophageal halitosis; 4—blood-born halitosis; and 5—subjective halitosis, which is further divided into halitosis of psychologic or neurogenic background [4].

The oral cavity is responsible for approximately 90% of halitosis cases [7]. Among oral problems such as gingivitis, periodontitis, dental caries, oral ulceration or food impaction, the tongue coating is considered to be the main cause of halitosis; however, the exact microbiological causative factors remain unclear [7]. Extra-oral causes of halitosis are responsible for approximately 5–10% of all malodor cases and ear/nose/throat, respiratory diseases or foreign bodies in the airway are the most common among them. Specific chronic diseases (such as gastric reflux, diabetes, liver or kidney disease), drugs (e.g., paracetamol, chloral hydrate, dimethyl sulfoxide, disulfiram, nitrate and nitrites, amphetamines) may also result in halitosis [7]. Different psychologic and neurogenic factors can cause pathologic subjective halitosis, which is known as a malodor complaint without objective confirmation by others or by measurements [8]. Most subjective halitosis cases are due to psychological factors [8]. It has been reported to be associated with conditions such as social anxiety disorder, obsessive compulsive spectrum disorder, stress, depression and olfactory reference syndrome (ORS)—preoccupation with the false belief that one emits a foul and offensive body odor accompanied by shame, embarrassment, significant distress, avoidance behavior and social isolation [8]. Patients with ORS are concerned with multiple body parts; however, the oral cavity is the most common one. Among neurogenic causes of pathologic subjective halitosis, chemosensory disorders such as olfaction and gustation are among the most common [8].

It is important to mention that the diagnostic distinction between objective and subjective halitosis may not be easy as sometimes patients are in between these two conditions: when objective halitosis has not been treated, it may cause the patient’s over-concern with the malodor to develop. In such a situation, after the breath odor is reduced to physiologic levels, the negative concern may persist, making the halitosis difficult to treat. On the other hand, oversensitivity to physiologic odor may be the basis of a subjective halitosis with no history of objective one [4].

The oral production of malodorous substances is most commonly associated with the by-products of bacterial metabolic degradation [1]. These products result from the microbial fermentation of proteins, peptides and mucins [1]. The most malodorous compounds are volatile sulfur compounds (VSCs), among which hydrogen sulfide (H_2_S), methyl mercaptan (CH_3_SH) and dimethyl sulfide ((CH_3_)_2_S) are 90% components of intra-oral halitosis [1]. Direct breath odor diagnostic methods include the organoleptic method, gas chromatography (GC) or sulfide monitoring. By using a sulfur detector, GC can specifically detect each VSC [1].

Halitosis is an underestimated problem affecting people globally. It has a significant impact on QOL and can result in psychological consequences. The phrase “bad breath” has a negative meaning and is associated with stigma. Patients with halitosis have a conviction that they have a problem, which can be easily noticed by others. This fact evokes feelings of low self-esteem, social and professional withdrawal, constant thoughts of having breath odor and interpreting gestures as disgust related to their malodor. These patients may also experience behavioral changes such as talking less [1,6]. Social anxiety disorder has been identified as the most common psychopathology associated with halitosis. It usually has an early onset and serious effects on social interactions and QOL [9]. It was found that patients seek a solution to the problem of halitosis precisely because of embarrassment and social harm [10].

There are not many descriptions of the assessment methods of QOL in patients with halitosis. Some authors have claimed that due to the large number of patients with subjective halitosis, the questionnaires are useless and lead to misdiagnosis [11]; however, it seems that they are a useful diagnostic tool in planning a comprehensive multidisciplinary approach as they allow to assess patients’ needs as well as the impact of the therapy on QOL after the treatment [12]. Tanaka et al. proposed examining patients using a two-part questionnaire [13]: the Halitosis Associated Quality of Life Test (HALT), developed by Kizhner et al. [12]; and the Halitosis Consequences Inventory (HCI), developed by Conceicao et al. [3]; however, there are no studies investigating the direct correlation between diagnosed intra-oral halitosis and all components of health.

Short Form 36-item Health Survey (SF-36) is a multi-purpose, short-form health survey [14]. The questionnaire allows the evaluation of QOL in eight domains: physical fitness (PF); activity limitations due to physical problems (RP); pain complaints (BP); general health perception (GH); vitality (VT); social functioning (SF); mental health—emotional wellbeing (MH); and activity limitations caused by emotional problems (RE) [15]. QOL in each of the domains is expressed with a number from 0 to 100. Higher numbers mean a better QOL. There are no standards for SF-36, so it cannot be said whether the results achieved by the respondents mean high or low QOL [15]. One can only compare domains with each other to identify the areas of best and poorest QOL [15]; however, attempts to use single SF-36 total score as the QOL measure have been made [16].

Considering all the above aspects, a hypothesis of the impact of intra-oral halitosis on patients’ QOL in both physical and mental components was conceived and an SF-36 questionnaire was applied to assess the domains of health.

### 1.2. Objectives

The aim of this study was to evaluate the mental and physical domains of health among patients with intra-oral halitosis and relevantly, investigate any differentiating factors. The main research concern was to assess whether intra-oral halitosis may impact patients’ QOL.

## 2. Materials and Methods

### 2.1. Trial Design

The trial was a single-center, prospective, clinical trial conducted at the Periodontology Department of University Dental Clinic in Cracow, Poland, between 7 January 2019 and 10 January 2020. This study was performed in accordance with the Declaration of Helsinki. All participants gave written informed consent to participate in this study. Official approval from the Jagiellonian University Ethics Committee was obtained (No. KBET/106/B/2011). A total of 167 patients complaining about oral malodor were checked for eligibility criteria during the first dental appointment. Among these, 67 did not meet the inclusion criteria (12 patients had general diseases, 33 patients had oral cavity diseases and in 22 cases, intra-oral halitosis was not confirmed by GC examination). Patients enrolled in the trial were asked to complete an anonymous questionnaire based on SF-36. They were then referred for follow-up care for halitosis.

### 2.2. Participants

One hundred generally healthy adult participants (58 females and 42 males; aged between 19 and 75 years; mean age 43.5 years) complaining about oral malodor, tongue coating and diagnosed with intra-oral halitosis were enrolled in this study. None of the participants had taken any antibiotics in the last 6 months and non-steroid anti-inflammatory drugs nor corticosteroids within the last 3 months. The patients had to be generally healthy with no diseases (including pf the respiratory and gastrointestinal tract, as well as metabolic disorders). They had to be nonsmokers, free from caries, oral potentially malignant disorders, inflammatory lesions of the oral mucosa, xerostomia, Sjögren’s syndrome and gingivitis or periodontitis. The patients had to have at least 20 teeth and not be using any prosthodontic or orthodontic appliances. Pregnancy, extended fasting, pharmacologic or radiologic therapy in the 6 months preceding the study were also added to the exclusion criteria.

### 2.3. Data Collection

Data were collected at baseline during the first scheduled dental appointment. Medical history, medication use, demographics, and oral hygiene routine were recorded. Oral examination was performed and intra-oral halitosis investigated. Tongue coating presence was assessed [17]. Patients were asked to complete an anonymous questionnaire. Clinical examinations were always performed in the morning. The patients were asked not to consume garlic, onion, spicy food and alcohol beverages one day before the examination and they were instructed not to use any scented personal products, not to eat breakfast, or brush their teeth on an appointment day.

### 2.4. Halitosis Assessment

A single trained examiner performed the clinical investigation. For halitosis diagnosis, a GC by Oral Chroma portable device (CHM-1, Abimedical, Abilit Corporation, Osaka City, Japan) was used according to the manufacturer’s instructions: 1.0 mL of the air sample was drawn from the headspace vial, 0.5 mL was expelled out, the remaining 0.5 mL was injected into the Oral Chroma and the readings were recorded. Sample collection was performed using a disposable syringe, which was inserted into the oral cavity. The patients were asked to breathe through their nose while keeping the oral cavity sealed and unventilated for at least 1 min. After one minute, the piston was pulled to the very end of the syringe and the syringe was filled again with a breath sample. The concentrations of hydrogen sulfide (H_2_S), methylmercaptan (CH_3_SH) and dimethyl sulfide ((CH_3_)_2_S) were measured in parts per billion (ppb). The total sum of all three VSCs higher than 146 ppb was considered as intra-oral halitosis (cognitive threshold set by the manufacturer for each gas: H_2_S = 112ppb; CH_3_SH = 26ppb; (CH_3_)_2_S = 8ppb) [5].

### 2.5. Questionnaire

The survey consisted of two parts. The first part comprised of demographic data, including age and sex. The second part of the questionnaire was based on the validated Polish version of SF-36 [18]. The questionnaire allows to assess the QOL in eight domains: physical fitness—PF (physical functioning); activity limitations due to physical problems—RP; pain complaints—BP; general health perception—GH; vitality—VT (energy/fatigue); social functioning—SF; mental health—MH (emotional wellbeing); and activity limitations caused by emotional problems—RE. The QOL in each of the domains is expressed with a number from 0 to 100. Higher numbers mean a better QOL. There are no standards for SF-36, so it cannot be said whether the results achieved by the respondents mean a high or low QOL. One can only compare domains with each other to identify the best and poorest areas of QOL [14].

### 2.6. Statistical Methods

Quantitative variables were summarized with the mean, standard deviation, median, quartiles and range. Qualitative variables were summarized with the number and percentage of occurrences for each possible value. The comparison of quantitative variables in two groups was performed with a *t*-test (if the variables were normally distributed in both groups) or with the Mann–Whitney test (otherwise). In order to obtain the effect size of 0.5 for the determination of the difference in numerical variables between the measurements, with the level of significance set to 0.05, the calculated minimum required sample size was 94 subjects. Correlation between two quantitative variables was assessed with the Pearson correlation coefficient (if both variables were normally distributed) and with Spearman rank correlation coefficient (otherwise). The strength of the relationship was interpreted as follows: |r| ≥ 0.9—very strong correlation; 0.7 ≤ |r| < 0.9—strong correlation; 0.5 ≤ |r| < 0.7—moderate correlation; 0.3 ≤ |r| < 0.5—weak correlation; |r| < 0.3—very week correlation according to interpretation schema by Hinkle et al. [19]. Cronbach’s alpha test was used to measure the SF-36 Survey reliability. Normality was checked with the Shapiro–Wilk test. The significance level was set to 0.05. All calculations were performed in the R package, version 3.4.2 (R Core Team (2013). R: A language and environment for statistical computing. R Foundation for Statistical Computing, Vienna, Austria).

## 3. Results

### 3.1. Adverse Events and Safety Monitoring

No adverse events were tracked and no rescue therapy was required for any of the patients throughout this study. All patients enrolled in this study were followed for halitosis after data collection.

### 3.2. Study Population

A total of 100 individuals with diagnosed halitosis participated in this study. The description of the study population is presented in Table 1.

### 3.3. SF-36 Domains’ Correlations

**Figure 1:**
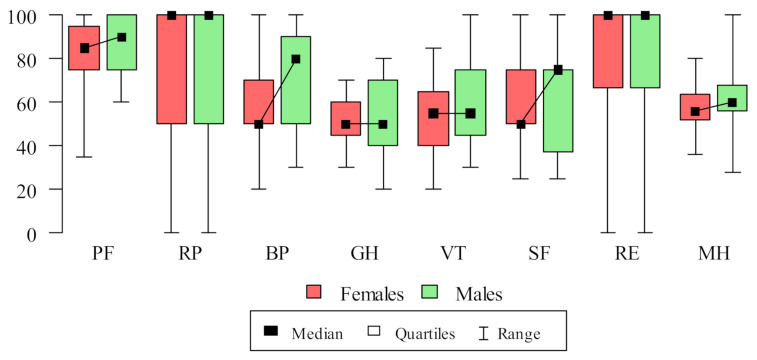
Differences between SF-36 domains between sexes.

The results of the SF-36 questionnaire are presented in Table A1. The reliability level (Cronbach alpha) of all eight scales was higher than 0.80 for each one with the median 0.85. The respondents had the highest scores in the PF (mean 83.8; SD = 15.96); RE (mean 75.33; SD = 34.21); RP (mean 70; SD = 38.8); and BP (mean 64; SD = 24.33) domains, and the weakest in the GH (mean 51.5; SD = 13.49); VT (mean 56; SD = 17.11); MH (mean 59.84; SD = 13.64); and SF (mean 61.5; SD = 22.29) domains. When analyzed by gender, SF-36 data were non-normally distributed, so an analysis with the Mann–Whitney test was conducted and presented in Figure 1. Only BP domain results were statistically significant and they were higher for males (mean 73.33; SD = 24.97 versus 57.24; SD = 21.86 among females; *p* = 0.024).

**Figure 2:**
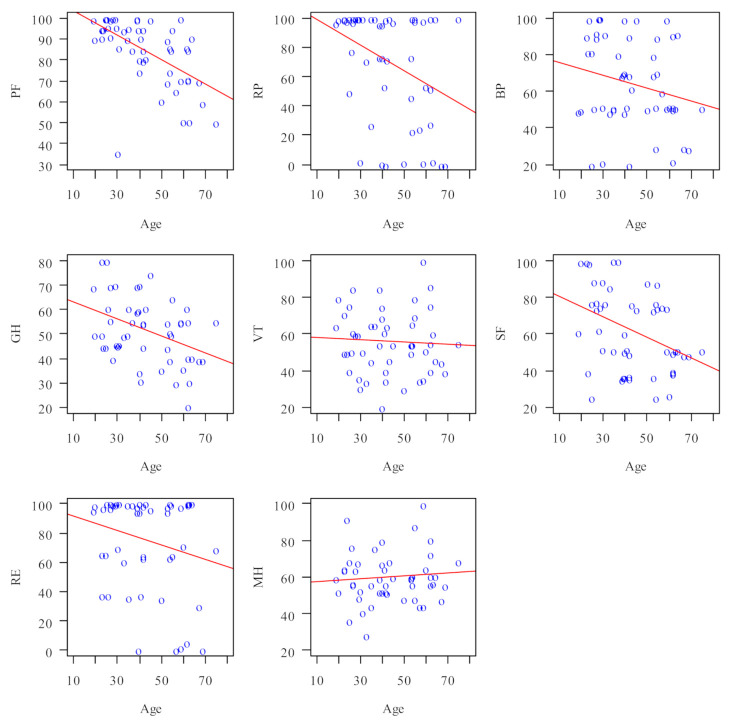
SF-36 domains and age correlation (age in years).

With regard to the SF-36 results and age, the data were non-normally distributed, so the Spearman correlation coefficient was applied for analysis and the data are presented in Figure 2 and Table 2. The strongest relationship was observed between age and PF (physical functioning; *p* < 0.001). There was a negative correlation between age and the PF (physical functioning; *p* < 0.001), RP (activity limitations due to physical problems; *p* = 0.015), GH (general health perception; *p* = 0.008) and SF (social functioning; *p* = 0.009) domains—the scores decreased with age.

The SF-36 and H_2_S levels’ data were non-normally distributed, so the Spearman correlation coefficient was applied for analysis and the results are presented in Table 3. The H_2_S level was negatively correlated with all SF-36 domains. The SF-36 domains’ scores decreased the higher the level of H_2_S was. The strongest correlation was observed between SF (−0.646; *p* = 0.005) and VT (−0.601; *p* = 0.003) compounds.

The SF-36, CH_3_SH and (CH_3_)_2_S data were non-normally distributed, so the Spearman correlation coefficient was applied for analysis and the data are presented in Table 4 and Table 5. There was no statistically significant correlation between those variables.

The SF-36 results and VSC scores were non-normally distributed, so the Spearman correlation coefficient was applied for analysis and the data are presented in Table 6. The VSC level was negatively correlated with all SF-36 domains. The SF-36 domains’ scores decreased the higher the level of VSCs was. The strongest correlation was observed between the SF (−0.642) and VT (−0.631) compounds.

## 4. Discussion

QOL has become a significant concept for practice and research in the field of health and the use of its assessments has increased [20]. Analyzing QOL is not only very important for improving patient care and rehabilitation but also for modifications in treatment as it may help identify a wide spectrum of problems affecting patients [21]. QOL evaluation is also a predictor of therapy success and a strong predictor of survival [20]. The World Health Organization (WHO)’s definition of QOL is “individuals’ perception of their position in the life in the context of the culture in which they live and in relation to their goals, expectations, standards and concerns” [22] and the health-related quality of life (HRQOL) is described as “a term referring to the health aspects of quality of life, generally considered to reflect the impact of disease and treatment on disability and daily functioning; it has also been considered to reflect the impact of perceived health on an individual’s ability to live a fulfilling life.” [23]. QOL is a complex concept defined in a number of ways and as a consequence, many different instruments are now used in its assessment [21].

The SF-36 is a multi-purpose health survey which yields a profile of functional health and wellbeing. It is a generic tool, opposed to ones that target a specific age, disease or treatment group [24]. The usefulness of the SF-36 in estimating disease burden and comparing disease benchmarks with general population norms is described for more than 200 diseases and conditions [25]. Among the most frequently studied diseases and conditions are arthritis, back pain, cancer, cardiovascular disease, chronic obstructive pulmonary disease, depression, diabetes, gastro-intestinal disease, migraine headache, HIV/aids, hypertension and irritable bowel syndrome, psychiatric diagnoses, surgical procedures and trauma. Translations of the SF-36 have been the subject of more than 500 publications [25]. Results from clinical studies comparing scores for patients before and after treatment have largely supported hypotheses concerning the validity of SF-36 scales [25]. For example, clinical investigations have shown that three of the scales (PF, RP and BP) with the most physical factor content tend to be the most responsive to the benefits of knee replacement [26] or heart valve surgery [27]. In contrast, the three scales with the most mental factor content (MH, RE and SF) in analytic studies have been shown to be the most responsive in comparisons of patients before and after recovery from depression [28] as well as drug treatment and interpersonal therapy for depression [29]. Although intra-oral halitosis can impact patients’ QOL, this relationship has not been largely evaluated by generic tools such as SF-36 [30]. The most frequently used surveys for QOL assessment in patients with halitosis are OHIP-14 and HALT [12,14,31,32], which do not estimate the full aspects of functional health and wellbeing as well as mental components of health at the same time.

In current study, among all SF-36 domains, the respondents diagnosed with intra-oral halitosis scored the highest in physical functioning and activity limitations caused by emotional and physical problems, means that they assessed their QOL best in these components of health and did not feel their functioning and activity distracted as much as general health perception, vitality and emotional wellbeing. Similar conclusions were found in a study by Lu at al. [31]. The authors compared the differences in Oral Health Related Quality of Life (OHRQOL) between halitosis and non-halitosis patients by the use of OHIP-14. The halitosis group had significantly higher OHIP-14 scores than the control group, which means that a poorer QOL and the most commonly reported negative impacts were also within the domains of ‘psychological discomfort’ and ‘psychological disability’. The same questionnaire (OHIP-14) was also used in an analysis by Buunk-Werkhoven et al. [32]. The authors indicated that the treatment of intra-oral halitosis plays an important role in self-perceived OHRQOL, especially in the psychological comfort aspect.

The sex differences analysis of the current study revealed the only statistically significant correlation in terms of pain complaints. Males assessed the QOL in this domain much better than women. In recent years, the number of studies regarding sex differences in pain have increased [33]. The literature reviews suggest that males and females differ in their responses to pain, with increased pain sensitivity observed among females [34]. Although the specific etiological basis underlying these differences is unknown, it seems that multiple biological and psychosocial processes are contributing factors [33]. Evidence suggests that genotype, endogenous opioid functioning, and sex hormones may influence pain sensitivity [35]. Additionally, psychosocial processes such as pain coping and early life exposure to stress as well as stereotypical gender roles may be a contributing factor [36]. Considering all these aspects, these results in the pain complaints domain do not seem to be surprising.

The analysis revealed a strong negative correlation between QOL and the age of patients with intra-oral halitosis. The scores decreased with age, especially in terms of physical functioning, pain complaints, general health perception and social functioning. Self-reported QOL usually follows a U-shaped or curvilinear relationship over the course of the life cycle with the lowest points reported when a person was between their mid-30s and early 50s [37]. Possible explanations for a lower QOL in midlife include the deterioration of health, accumulated life stressors, and demands from work and family affecting wellbeing [38]. Considering the fact that the mean age of the investigated population was 43.5 years, the outcomes of the current study are similar to other findings. The same results regarding the QOL and age relation were observed in many investigations. Kilkenny et al. [39] concluded that age appears to be a key determinant of HRQOL and Lannin et al. [40] demonstrated a trend between increasing age and decreasing self-reported overall health status with the average midlife age of the investigated group. On the other hand, findings by Tseng et al. [40] indicated that in spite of objective health decrements, subjective OHRQOL is maintained among elderly Chinese subjects [41], confirming the U-shaped relation between age and life quality.

Although negative, there was no statistically significant correlation between the SF-36 results, CH_3_SH and (CH_3_)_2_S but the H_2_S and VSC levels were significantly negatively correlated with all domains (the higher the level of VSCs was, the more the SF-36 domains’ scores decreased). Considering this fact and statistical analysis, it seems that H_2_S was the main sulfur compound influencing the overall outcomes of the current study. As a cognitive threshold was set by the GC manufacturer for H_2_S (112 out of 146 ppb for total VSCs) for intra-oral halitosis diagnosis, these findings are within expectations. H_2_S and mercaptans are the principal end-products of the metabolism of sulfur amino acids: methionine, cysteine and homocysteine in the Gram-negative anaerobic bacteria [42,43]. The amount of H_2_S in saliva among healthy individuals was reported to be 1.641–7.124 µM [43], whereas in patients with intra-oral halitosis, it achieved a 6.7 ng/10 mL concentration [44].

As the QOL decreased with halitosis (VSCs) levels, the strongest correlation was found in the social functioning, vitality, emotional wellbeing and general health perception domains. Similar outcomes regarding different aspects of QOL were found in several studies with different methodology. In a study by Conceicao et al., the social anxiety disorder assessment of individuals with halitosis revealed the desire to avoid being with or talking to other people, in addition to reporting feelings of being upset, tense or anxious during social interactions. These feelings were more likely to be especially present among individuals with a strong belief that others were noticing their breath odor [3]. The assessment of the impact of intra-oral health on QOL by Santaella et al. [45] showed a negative influence of halitosis on social disability and psychological discomfort. According to Troger et al. [46], halitosis may cause embarrassment, depression and make relationships more difficult. In this study, 104 patients (out of 274) felt tense, 192 felt ashamed, 85 had difficulty smiling, 198 did not feel comfortable talking to others and 82 reported difficulties in dating. The authors concluded that the psychological condition of the patients might be related to the degree of halitosis and their clinical characteristics. A Brazilian study also found a high prevalence of psychological conditions in patients with halitosis as they reported higher levels of depression, avoidance behaviors as well as poor self-care. The authors indicated that freedom from disabling breath odor is an outcome indicator of social wellbeing [47]. In another study, Suzuki et al. evaluated that the psychological condition of patients complaining of halitosis was associated with the actual degree of malodor and the clinical characteristics [48]. Data from a study by Azodo et al. revealed evident social distance towards halitosis sufferers, usually in the form of hesitance to talk to others, feeling uneasy when someone is nearby, dislike meeting people and maintaining a distance [49]. According to another study, patients with breath odor exhibited a greater level of inadequacy, depression, anxiety, sensitivity, anger, stress and the psychological status of the patients varied with sex [50]. Veeresha et al. concluded that overall halitosis is a crippling social problem [51].

The latest systemic review and meta-analysis by Schertel Cassiano et al. [52] reveals that although the findings suggest that halitosis is associated with impaired oral health-related quality of life, there have not been many investigations regarding the impact of halitosis on QOL. In order to perform a systematic review of the literature, the authors performed electronic searches in PubMed via Medline, Web of Science, Scopus and EMBASE. They included thirteen studies in the review but the meta-analysis only included 10 studies. Although halitosis can impact QOL, this relationship has not been widely evaluated, especially by tools estimating the functional health and wellbeing components of health at the same time [30].

The present study has some limitations. Although the authors dedicated significant efforts to narrowing the inclusion criteria and maintaining the homogeneity of the study population, the results of the investigation might be interfered by multifactorial background of halitosis. Additionally, some other factors, not investigated in this study, such as halitosis duration and previous halitosis treatments’ results, could also influence patients’ QOL. Finally, QOL assessment only relies on self-perceived data, which are a strong subjective component in the way answers have been resolved by the patient.

Despite the limitations of this investigation, the significant influence of the intra-oral halitosis level on patients’ QOL was demonstrated—especially for the wellbeing components of health. As halitosis sufferers experience psychological consequences of their condition, future guidelines on complex health status assessments should be addressed by relevant institutions to provide protocols for multidisciplinary approach.

## 5. Conclusions

Within the limits of this study, the presented data indicate that the respondents assessed their QOL to be the worst with regard to general health perception, vitality and emotional wellbeing. This study revealed a strong negative correlation between QOL and the age of patients with intra-oral halitosis. The scores decreased with age, especially in terms of pain complaints, general health perception and social functioning. The SF-36 domains’ scores decreased with the higher intra-oral halitosis (VSCs) levels, the strongest correlation between intra-oral halitosis and decreased quality of life was found in the social functioning, vitality, emotional wellbeing and general health perception domains. Patients with intra-oral halitosis often experience psychological consequences and poor QOL, thus this condition requires not only professional care provided by dental specialists, but also a multidisciplinary and psychological support.

## Figures and Tables

**Table 1:** The description of the study population.

		Mean (SD)	Median (Quartiles)
H_2_S		496.22 (441.19)	412 (132.75–724.25)
CH_3_SH		205.66 (329.18)	53 (26–265.25)
(CH_3_)_2_S		147.36 (231.83)	40 (8–214)
Total VSC		849.24 (600.36)	761 (405.5–1246.75)
Age		43.5 (15.17)	41.5 (30–56.5)
		***N***	**%**
Sex	Female	58	58%
	Male	42	42%

**Table 2:** SF-36 domains and age correlation.

Domain	Correlation with Age			
	Correlation Coefficient	*p*	Direction	Strength [19]
PF (physical functioning)	−0.611	<0.001	Negative	Moderate
RP (activity limitations due to physical problems)	−0.341	0.015	Negative	Weak
BP (pain complaints)	−0.219	0.126	---	---
GH (general health perception)	−0.37	0.008	Negative	Weak
VT (vitality)	−0.105	0.469	---	---
SF (social functioning)	−0.368	0.009	Negative	Weak
RE (activity limitations caused by emotional problems)	−0.175	0.225	---	---
MH (emotional wellbeing)	0.028	0.849	---	---

---not statistically significant, *p* > 0.05.

**Table 3:** SF-36 domains’ and H_2_S correlation.

Domain	* Correlation with H_2_S			
	Correlation Coefficient	*p*	Direction	Strength [19]
PF	−0.332	0.015	Negative	Weak
RP	−0.613	0.511	---	---
BP	−0.352	0.008	Negative	Weak
GH	−0.386	0.009	Negative	Weak
VT	−0.601	0.003	Negative	Moderate
SF	−0.646	0.005	Negative	Moderate
RE	−0.312	0.015	Negative	Weak
MH	−0.512	0.04	Negative	Weak

* Spearman correlation; ---statistically not significant, *p* > 0.05.

**Table 4:** SF-36 domains’ and CH_3_SH correlation.

Domain	* Correlation with CH_3_SH			
	Correlation Coefficient	*p*	Direction	Strength [19]
PF	0.048	0.739	---	---
RP	0.047	0.747	---	---
BP	−0.032	0.827	---	---
GH	−0.067	0.643	---	---
VT	−0.244	0.087	---	---
SF	0.107	0.459	---	---
RE	−0.06	0.679	---	---
MH	−0.027	0.853	---	---

* Spearman correlation; ---statistically not significant, *p* > 0.05.

**Table 5:** SF-36 domains’ and (CH_3_)_2_S correlation.

Domain	* Correlation with (CH_3_)_2_S			
	Correlation Coefficient	*p*	Direction	Strength [19]
PF	0.094	0.518	---	---
RP	−0.159	0.269	---	---
BP	−0.01	0.942	---	---
GH	−0.062	0.667	---	---
VT	−0.023	0.876	---	---
SF	−0.065	0.655	---	---
RE	−0.206	0.151	---	---
MH	−0.268	0.06	---	---

* Spearman correlation; ---statistically not significant, *p* > 0.05.

**Table 6:** SF-36 domains’ and total VSC correlation.

Domain	* Correlation with Total VSC			
	Correlation Coefficient	*p*	Direction	Strength [19]
PF	−0.256	0.016	Negative	Very weak
RP	−0.289	0.001	Negative	Very weak
BP	−0.376	0.005	Negative	Weak
GH	−0.378	0.009	Negative	Weak
VT	−0.631	0.008	Negative	Moderate
SF	−0.642	0.015	Negative	Moderate
RE	−0.359	0.015	Negative	Weak
MH	−0.386	0.009	Negative	Weak

* Spearman correlation.

## Data Availability

This data presented in this study are available on request from the corresponding author. This data are not publicly available due to ethical restrictions.

## Appendix A

**Table A1:** Mean SF-36 results in the study group.

Domain	N	Mean	SD	Median	Min	Max	Q1	Q3
PF (physical functioning)	100	83.8	15.96	90	35	100	75	95
RP (activity limitations due to physical problems)	100	70	38.8	100	0	100	50	100
BP (pain complaints)	100	64	24.33	60	20	100	50	90
GH (general health perception)	100	51.5	13.49	50	20	80	41.25	60
VT (vitality)	100	56	17.11	55	20	100	45	65
SF (social functioning)	100	61.5	22.28	56.25	25	100	40.62	75
RE (activity limitations caused by emotional problems)	100	75.33	34.21	100	0	100	66.67	100
MH (emotional wellbeing)	100	59.84	13.64	60	28	100	52	67
